# Targeted CRM197-PEG-PEI/siRNA Complexes for Therapeutic RNAi in Glioblastoma

**DOI:** 10.3390/ph4121591

**Published:** 2011-12-16

**Authors:** Sabrina Höbel, Chantal C.M. Appeldoorn, Pieter J. Gaillard, Achim Aigner

**Affiliations:** 1 Institute of Pharmacology/Biochemical-Pharmacological Center (bpc), Faculty of Medicine, Philipps-University Marburg, Karl-von-Frisch-Strasse 1, D-35032 Marburg, Germany; 2 Rudolf-Boehm-Institute of Pharmacology and Toxicology, Clinical Pharmacology, Härtelstrasse 16-18, D-04107 Leipzig, Germany; 3 to-BBB technologies BV, Bio Partner Center II, Niels Bohrweg 11, 2333 CA Leiden, The Netherlands; E-Mail: PieterGaillard@toBBB.com (P.J.G.)

**Keywords:** siRNA, RNA interference, RNAi, CRM197, HB-EGF, polyethylenimine, PEI, targeted delivery, pleiotrophin, glioblastoma, therapeutic siRNA delivery

## Abstract

RNA interference (RNAi) allows the specific knockdown of tumor relevant genes. To induce RNAi, the delivery of small interfering RNAs (siRNAs) is of crucial importance. This is particularly challenging for their therapeutic applications *in vivo*. Low molecular weight branched polyethylenimine (PEI) is safe and efficient for nucleic acid delivery including small RNA molecules, based on its ability to electrostatically complex siRNA molecules, thereby protecting them from nuclease degradation. The nanoscale PEI/siRNA complexes are endocytosed by cells prior to intracellular complex release from the lysosome and cytoplasmic release of the siRNAs from the complexes. Chemical modification and ligand decoration of the complexes aim at introducing target tissue specificity and further increased efficacy of PEI-mediated siRNA delivery. CRM197 is a mutated, non-toxic diphtheria toxin (DT) that binds to the membrane-bound precursor of HB-EGF-like growth factor/diphtheria toxin receptor highly expressed in glioblastoma cells. Likewise, the growth factor pleiotrophin (PTN/HB-GAM/HARP) is overexpressed in glioblastoma and is rate limiting for tumor growth, thus representing an attractive target gene for therapeutic knockdown approaches. PEGylation of PEI was performed to reduce the surface charge, and by CRM197 coupling we prepared a modified PEI for siRNA delivery into glioblastoma cells. The novel PEI conjugates were analyzed for their complexation efficiency and optimal mixing ratios, and complexes were physicochemically characterized regarding stability, size and zeta potential. The biological activity of the complexes was confirmed in cell culture by reporter gene knockdown. For the therapeutic treatment of subcutaneous human gliobastoma xenografts in athymic nude mice, we systemically injected the modified PEI/siRNA complexes targeting PTN. Antitumor effects based on PTN knockdown demonstrated the advantage of tumor-targeted CRM197-PEG-PEI/siRNA over untargeted PEG-PEI polyplexes. Thus, we establish targeted CRM197-PEG-PEI-based complexes for siRNA delivery *in vivo*, and show therapeutic effects of CRM197-PEG-PEI/siRNA-mediated knockdown of PTN.

## 1. Introduction

RNA interference (RNAi) [1] is a naturally occurring mechanism of eukaryotic cells. It is believed to act as defense mechanism and to support the maintenance of the genome integrity. By delivery of the effector molecules of RNAi, the small interfering RNAs (siRNAs) [2], into cells, this mechanism can be activated and thus used for therapeutic knockdown [3] of any target gene of interest. Since the crucial step for the induction of RNAi is the delivery of intact siRNAs, formulations based on viral or non-viral vectors, especially various polymer and lipid based delivery systems, have been described and are superior to the administration of naked siRNA molecules. Non-viral delivery systems are less efficient, but show substantially reduced safety problems. Thus, different approaches and modifications of these delivery systems are investigated to produce efficient and cell-specific vectors.

Among cationic polymers, polyethylenimine (PEI) is one of the most efficient compounds for nucleic acid delivery [4,5]. PEI combines the properties of nucleic acid complexation and condensation, thereby preserving them from nuclease degradation. The formation of positive charged polyplexes allows cellular uptake and, due to the so-called “proton sponge” effect of PEI [6], the polyplexes are able to escape from the endolysosomal compartment. Once in the cytosol, siRNA molecules can be incorporated into the RNA induced silencing complex (RISC) to induce RNAi, and DNA or PEI/DNA-complexes can enter the nucleus for gene expression. Since its introduction as transfection reagent for DNA *in vitro*, PEI has been shown to be also highly efficient for administration of small nucleic acids like antisense ODNs, ribozymes, siRNAs and microRNAs [4,7,8,9,10]. Linear and branched PEIs with different molecular weights are available, with high molecular weight PEIs being more efficient but also more cytotoxic. Therefore, the low molecular weight branched PEI, PEI F25-LMW, generated by fractionation of 25 kDa HMW-PEI [11], has been used. Because of its low cytotoxicity and high biological activity, it proved to be a very useful delivery system for the transfection of nucleic acids *in vitro* and for the delivery of small RNAs *in vivo* [9,10].

One interesting target molecule for the treatment of defined tumor entities is the secreted growth factor pleiotrophin (PTN), also known as HB-GAM (heparin biding growth associated molecule) or HARP (heparin affinity regulatory peptide). PTN can act by binding to its receptor ALK (anaplastic lymphoma kinase) [12], and is essential for the growth of neuronal cells and for proliferation of fibroblasts, epithelial and endothelial cells [13]. Under pathological conditions, PTN promotes the growth of various tumor cells including glioblastomas, and serves as angiogenic and metastasis promoting factor [14,15]. In previous studies, it has been shown that PTN is highly expressed in tumor cell lines of different origin [16-19] and that knockdown of PTN results in reduced growth of glioblastoma xenografts [20-22].

For the targeted delivery of the PEI/siRNA complexes, we chemically modified the polymer in order to develop nanoparticles with altered physicochemical and biological properties, altered pharmacokinetics and tissue- or cell-specific uptake of the polyplexes. For the generation of a glioblastoma specific formulation, we coupled a ligand of the diphtheria toxin receptor (DTR) to PEI F25-LMW. The diphtheria toxin receptor is constitutively expressed on endothelial cells, including on those forming the blood-brain barrier, neurons and glial cells [23]. The strong upregulation of the DTR in gliomas [24], however, is expected to lead to a site-selective improvement of the therapeutic efficacy of siRNA-mediated knockdown of PTN. Since diphtheria toxin, the extremely toxic ligand for DTR, cannot be used for therapeutic purposes, the non-toxic mutant CRM197, which has been used for a long time as carrier protein in human vaccines [25], was developed for drug targeting [26,27]. It is known that CRM197 binds to the membrane-bound precursor of DTR [23] and is internalized by receptor-mediated endocytosis [28]. To reduce unspecific cellular uptake of the positively charged polyplexes, we performed PEGylation of PEI and conjugation of CRM197 to PEI via a PEG spacer.

In this study we have optimized the complexation efficiency of CRM197-PEG-PEI as well as the physicochemical properties necessary for efficient nucleic acid delivery. The efficacy of the CRM197-PEG-PEI/siRNA complexes was investigated *in vitro* using a luciferase activity assay. Finally, the anti-tumor efficacy of CRM197-PEG-PEI/PTN was determined in mice with subcutaneous human glioblastoma xenografts, and compared to (non-targeted) PEG-PEI/PTN.

## 2. Materials and Methods

### 2.1. siRNAs, Tissue Culture and Animals

Chemically synthesized siRNA duplexes directed against luciferase (pGL3 and pGL2 as a negative control) were purchased from MWG (Ebersberg, Germany). SiRNA against PTN with passenger strand sequence 5′-GGAAGGCAAGAAACAGGAGdTdT-3′ and guide strand sequence 5′-CUCCUGUUUCUUGCCUUCCdTdT-3′ were obtained from Ambion/Applied Biosystems (Darmstadt, Germany).

U87 glioblastoma cells were obtained from the American Type Culture Collection (ATCC/LGC Promochem, Wesel, Germany) and cultivated in a humidified incubator under standard conditions (37 °C, 5% CO_2_) in IMDM (PAA, Cölbe, Germany) supplemented with 10% fetal calf serum (FCS).

Athymic nude mice (nu/nu) were purchased from Harlan Winkelmann (Borchen, Germany) and kept at 23 °C in a humidified atmosphere with food and water *ad libidum*. Animal studies were approved by the Regierungspräsidium Giessen, Germany.

### 2.2. Conjugation of CRM197 to PEI and Preparation of PEI/DNA and PEI/siRNA Complexes

Polyethylenimine, PEI F25-LMW, was prepared as described previously [11]. Briefly, 100 mg 25 kDa PEI (Al 25-kDa, Sigma-Aldrich, Taufkirchen, Germany) was fractionated by size exclusion chromatography on Sephadex G-50 fine resin (Amersham Biosciences, Freiburg, Germany) in 150 mM NaCl. One mL fractions 55–80 were collected in an automated fraction collector (Amersham Biosciences), pooled and concentrated in an Amicon stirred cell with an Amicon YM1 Ultrafiltration Disc, 1,000 NMWL (Millipore, Eschborn, Germany). For the determination of PEI concentrations, 100 μL/well 0.02 M cupric acetate in 5% potassium acetate (pH 5.5) and 20 μL of the sample or a PEI standard of known concentration were mixed in a 96-well plate. The absorption at 630 nm was measured using an ELISA reader (Bio-Tek Instruments, Winooski, VT), and concentrations were determined by comparison with the standard curve.

PEI F25-LMW was conjugated to CRM197 ([Glu^52^]-Diphtheria toxin, from *Corynebacterium diphtheria*, Sigma-Aldrich, Taufkirchen, Germany) using the following procedure. First, PEI F25-LMW (5 mg/mL in PBS) was incubated for 1 h at room temperature with 2 eq. of NHS-PEG_5000_-VS (Nektar, Huntsville, AL, USA) generating PEI-PEG-VS, after which the excess of NHS-PEG_5000_-VS was removed with a Zeba column (Pierce, Rockford, IL USA). During this time CRM197 (3 mg/mL in 160 mM borate buffer, pH 8) was incubated with 10 eq. of SATA (Pierce, Rockford, IL, USA) according to manufactures instructions, after which the excess SATA was removed by passing the solution through a Zeba column. The activated CRM197 contained 1–2 -SH groups (data not shown) and was immediately incubated with PEI-PEG-VS using a 1:1 molar ratio for 18 h at 4 °C. The CRM197-PEG-PEI was stored at 4 °C until use. As a control, PEI-PEG was prepared by quenching PEG-PEI-VS with 10 eq. of mercaptoethanol, and subsequent removal of the excess of mercapto-ethanol.

For PEI complexations, the optimal PEI/nucleic acid (*i.e.*, DNA or siRNA) ratios were determined, which are expressed as PEI/DNA or PEI/RNA equivalents (N/P ratios) on the basis of PEI nitrogen per nucleic acid phosphate (1 μg of DNA or siRNA is 3 nmol of phosphate, and 1 μL 1 mg/mL PEI solution contains 23 nmol of amine nitrogen). 0.5 μg nucleic acids (DNA or siRNA) were dissolved in 40 μL 150 mM NaCl buffered with 10 mM HEPES, pH 7.4, and in a separate vial appropriate amounts of PEI solution (5 μg PEI F25-LMW = N/P 77) were dissolved in 40 μL of the same buffer. After 10 min, the PEI solution was pipetted into the DNA or siRNA solution, respectively, resulting in the desired N/P ratio. The mixture was vortexed, incubated for 30 min at room temperature, vortexed again and added to the cell culture medium. For *in vivo* experiments, 10 μg siRNA and 150 μg PEG-PEI or CRM197-PEG-PEI were dissolved in 75 μL 150 mM NaCl buffered with 10 mM HEPES, pH 7.4, in separate vials prior to mixing the solutions as described above.

### 2.3. Transfection of Cells and Determination of Luciferase Activity

For transfection experiments, complexes were prepared according to the procedure described above. U87 glioblastoma cells were seeded at 4 × 10^4^ cells/well in 24-well plates and PEI/DNA complexes containing 0.5 μg DNA were added to the cell culture medium of each well. 24 h after DNA transfection luciferase knockdown was induced by cotransfection of the cells with PEI/siRNA complexes containing 30 pmol luciferase siRNA GL3 (specific) or GL2 (unspecific). After cultivation of the cells for 48 h, luciferase activity was determined using the luciferase assay kit from Promega (Mannheim, Germany) according to the manufacturer's protocol. Briefly, the medium was aspirated and the cells were lysed in 100 μL lysis buffer. In a luminometer tube, 25 μL substrate was mixed with 10 μL lysate, and chemoluminiscence was determined immediately in a luminometer (Berthold, Bad Wildbad, Germany). In competition experiments, the peptide CRM197 was diluted in IMDM and added to the cells 30 min prior to the transfection with CRM197-PEG-PEI/siRNA complexes.

### 2.4. Determination of the Viability of Glioblastoma Cells Treated with CRM197

Cell viabilities in the presence of CRM197 were determined as described previously [29]. Briefly, 500 cells per well were seeded in 96-well plates and, after 24 h, different amounts of CRM197 diluted in IMDM were added to the cell culture medium. Numbers of viable cells were determined using a colorimetric assay, which is based on the cleavage of the tetrazolium salt WST-1 by mitochondrial dehydrogenases, according to the manufacturer's protocol (Cell Proliferation Reagent WST-1, Roche Molecular Biochemicals, Basel), with each value representing the mean of triplicate wells.

### 2.5. Determination of Complexation Efficiency of PEI Conjugates and Stability of PEI/siRNA Complexes

siRNA was [^32^P] end-labeled using T4 polynucleotide kinase according to standard protocols with 10 μg siRNA and 100 μCi γ-[^32^P]ATP. Purification was performed using Micro Bio-Spin Chromatography Columns (BioRad Laboratories, CA) by applying the solution onto pre-centrifuged (2 min, 1,000 × g) P-6 columns and subsequent centrifugation at 1,000 × g for 4 min for recovering the labelled nucleic acids in the flowthrough, and mixed 1:2 with unlabeled siRNA.

To determine the efficacy of PEI/siRNA complex formation, 200 ng siRNA in 15 μL complexation buffer (10 mM HEPES/150 mM NaCl, pH 7.4) were complexed with the corresponding PEI amounts as indicated in the figures. 1% agarose gel electrophoresis was performed at 80 mV for 2 h as described previously [11], and siRNAs were analysed by autoradiography in a Cyclone Plus Phosphoimager (Perkin-Elmer, Fremont, CA). Percentages of nucleic acid complexation were calculated based on the quantity of free siRNA relative to completely complexed (100%) samples. Relative stabilities of the complexes were determined in a polyanion competition assay by measuring the siRNA release from complexes in the presence of heparin [29]. [^32^P]-labelled siRNA was complexed as described above and treated with different amounts of heparin from 0.01 to 10 International Units (IU)/200 ng of siRNA in 24 μL HEPES/NaCl buffer over 15 min. Afterwards, samples were separated by electrophoresis and analyzed as described above.

### 2.6. Photon Correlation Spectroscopy (PCS) and Laser Doppler Anemometry (LDA)

The measurement of the hydrodynamic diameters and surface charge of PEI/siRNA complexes was essentially as described previously [30], with complexes prepared under the conditions described above. The polyplex sizes were determined by PCS using a Zetasizer 3000 HS from Malvern Instruments (Herrenberg, Germany) equipped with a 10 mW HeNe laser at a wavelength of 633 nm at 25 °C. Scattered light was detected at a 173° angle with laser attenuation and measurement position adjusted automatically by the Malvern software. Values are given as the means +/− standard deviation (SD) of three measurements of the sample with at least 10 runs each, as determined by the zetasizer. The zeta potential of the complexes was determined by LDA using a capillary electrophoresis cell of the Zetasizer Nano ZS at 25 °C, and the light signal detected at a 17° angle. Average values were calculated based on the data of three times 10 runs +/− SD.

### 2.7. PEI-Mediated siRNA Treatment of Subcutaneous Glioblastoma Xenografts in Mice

3 × 10^6^ U87 glioblastoma cells in 150 μL PBS were injected subcutaneously (s.c.) into both flanks of athymic nude mice (nu/nu). When solid tumors were established, mice were randomized into treatment groups and treated with 10 μg of PEI-complexed PTN-siRNA, administered by intraperitoneal injection, at the time points indicated in the figures. Tumor growth was monitored every 2–3 days, and tumor sizes were estimated by the formula ½(a·b^2^) with a = length and b = width of the tumor. Upon termination of the experiment, mice were killed and tumors were removed and snap frozen for ELISA measurements.

### 2.8. Pleiotrophin ELISA

For the determination of PTN serum levels, blood was collected from the heart and was centrifuged after coagulation at 13,000 rpm for 10 min. Supernatants were transferred to a fresh tube and samples were diluted in reagent diluent (1% BSA in PBS). PTN serum concentrations were determined using a specific PTN ELISA essentially as described previously [16].

Briefly, a mouse anti-PTN monoclonal antibody (4B7, 100 μL/well), diluted to 1 μg/mL in Tris-buffered saline (TBS), was incubated in covered 96-well ELISA plates (Life Technologies, Karlsruhe, Germany) at 4 °C overnight. Wells were washed three times with TBS/0.5% Tween 20 (TBST), blocked with 200 μL of TBST/1% bovine serum albumin (BSA) for 2 h, and washed again prior to the addition of the samples (100 μL/well). After incubation for 1 h and subsequent washing, biotinylated affinity-purified goat anti-human PTN detection antibody (100 μL/well; R&D Systems, Wiesbaden, Germany) was added at a concentration of 500 ng/mL and incubated for 1 h, washed again, and incubated with streptavidin-alkaline phosphatase conjugate (100 μL/well, diluted 1:5000 in TBST; Roche Diagnostics, Mannheim, Germany) for 1 h. After a final washing, the plate was incubated in the dark with p-nitrophenyl phosphate substrate solution (100 μL/well) for 2 h. Absorbance was measured in an ELISA reader at 405 nm with background subtraction at 595 nm. Recombinant human PTN (R&D Systems, Abingdon, UK) served as the standard.

### 2.9. Statistics

Statistical significance was analysed by Student's t-test or two-way ANOVA.

## 3. Results

**Figure 1:**
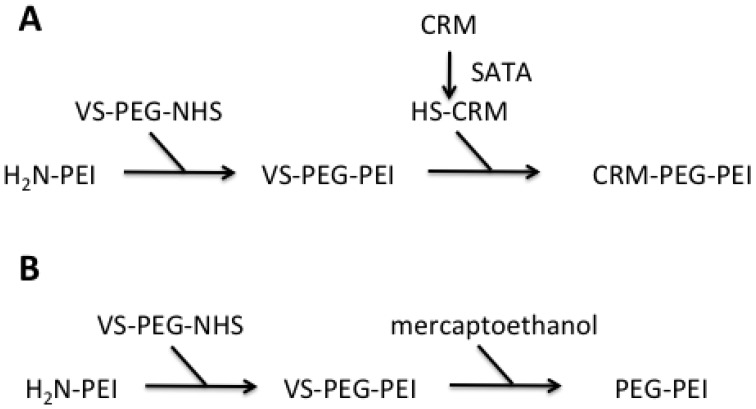
Schematic representation of the synthesis of PEI-conjugates. PEI F25-LMW was conjugated with VS-PEG-PEI to the intermediate VS-PEG-PEI. This intermediate was either transformed into CRM197-PEG-PEI via conjugation to SATA-activated CRM197 (**A**) or quenched with mercaptoethanol to obtain PEG-PEI (**B**).

Both PEI-conjugates were synthesized starting from PEI F25-LMW. Amine groups of the PEI were conjugated to bifunctional PEG spacer VS-PEG-NHS. This PEGylated PEI intermediate was subsequently reacted with an equimolar amount of CRM197, resulting in a 1:1:1 PEI-PEG-CRM197 conjugate [Figure 1(A)]. To be able to perform this conjugation, additional thiol groups were introduced to the CRM197 using SATA, a known amino acid modifier. For the sysnthesis of the control PEI-conjugate, the VS group of VS-PEG-PEI was quenched with an excess of mercaptoethanol [Figure 1(B)]The quantitative determination of CRM197 conjugated to PEI (measurement of A_280_ for protein quantitation, determination of PEI concentrations by a specific Cu-based assay) revealed a 0.5–2 molar ratio of CRM197:PEI, thus supporting the 1:1 molar ratio calculated.

**Figure 2:**
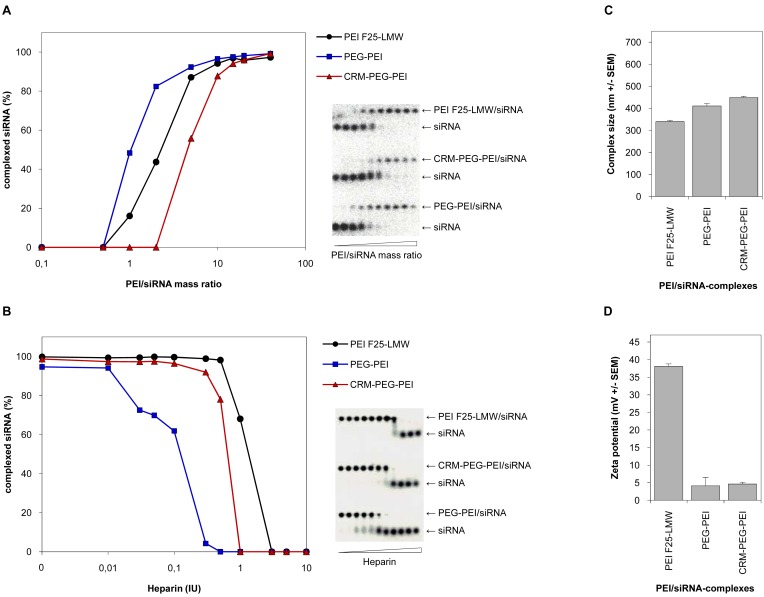
Characterization of PEI/siRNA complexes. (**A**) SiRNA complexation efficiencies of the parent PEI F25-LMW (black circles) and the novel PEI conjugates, PEG-PEI (blue rectangles) and CRM197-PEG-PEI (red triangles). (**B**) Stabilities of siRNA complexes against polyanions (heparin displacement assay). After incubation in the presence of various concentrations of heparin as indicated in the figure, complexes were analysed by agarose gel electrophoresis followed by autoradiography and quantitation of the ^32^P-labeled free or complexed siRNA (left panels; see right panels for original gels). (**C**) Hydrodynamic diameters of PEI/siRNA complexes as determined by photon correlation spectroscopy, and (**D**) surface charges (zeta potentials) of the complexes as determined by laser doppler anemometry.

To characterize the properties of the PEI-conjugates and to prove their applicability for nucleic acid delivery into cells, we investigated their complexation efficiency and physicochemical properties with regard to stability, size and surface charge of the polyplexes, and compared them to the parent polymer PEI F25-LMW. Only minor differences were observed between the siRNA complexation efficiencies of the chemically modified PEIs, PEG-PEI and CRM197-PEG-PEI, and PEI [Figure 2(A)]. At lower mixing ratios of polymer and nucleic acid, expressed as PEI/siRNA mass ratios, we detected a somewhat decreased complexation efficiency of PEG-PEI and a slightly higher complexation efficiency of CRM197-PEG-PEI compared to the unmodified PEI. Nevertheless, at higher mass ratios the complexation efficiencies of the parent polymer and the PEI-conjugates were similar, and at mass ratios 10 and higher, 90–100% of the siRNA molecules were complexed by all polymers. From these results, we concluded that at mass ratios ≥10 the ability of the PEI-conjugates to complex siRNA molecules was sufficient to generate polyplexes comparable to that of PEI.

By a heparin displacement assay we proved the integrity of the PEI/siRNA complexes in the presence of polyanions. To this end, siRNA molecules were complexed by PEI or the PEI-conjugates, respectively, at mass ratios where complete complexation of the nucleic acid is provided. This was mass ratio 5 for PEI and mass ratio 15 for the PEI-conjugates. The polyplexes were then exposed to different amounts of heparin as indicated in [Figure 2(B)]. We found a slightly decreased complex stability of CRM197-PEG-PEI compared to PEI, and for PEG-PEI a somewhat further decrease in the complex stability was detected. Still, we conclude that the complex stability of the PEI-conjugate CRM197-PEG-PEI is comparable to that of PEI and thus sufficient for the use of these complexes *in vivo*.

Since the size and the surface charge of polyplexes are critical parameters for cellular uptake, we next performed PCS and LDA measurements. The analysis of the hydrodynamic diameters of PEI/siRNA complexes showed minor differences regarding the size of the polyplexes. Compared to PEI/siRNA complexes, polyplexes with the PEI-conjugates showed an only slight increase in complex sizes. All polyplexes showed hydrodynamic diameters between 350 and 450 nm [Figure 2(C)]. Thus, conjugation of PEG or CRM197-PEG to PEI results in a small increase of the complex sizes. As expected, major differences were found regarding the surface charge of PEI/siRNA complexes. PEGylation of PEI or conjugation of CRM197 to PEI via a PEG-Spacer (Mw 5,000) resulted in a decrease of the zeta potential from 37 mV to 5 mV. No further reduction of the zeta potential was observed upon coupling of the ligand [Figure 2(D)]. This indicates that the PEGylation will lead to altered pharmacokinetics of the complexes in terms of prolonged serum halflives due to their reduced surface charge, while the ligand (CRM197) adds the tissue specificity without further alterations of the zeta potential.

**Figure 3:**
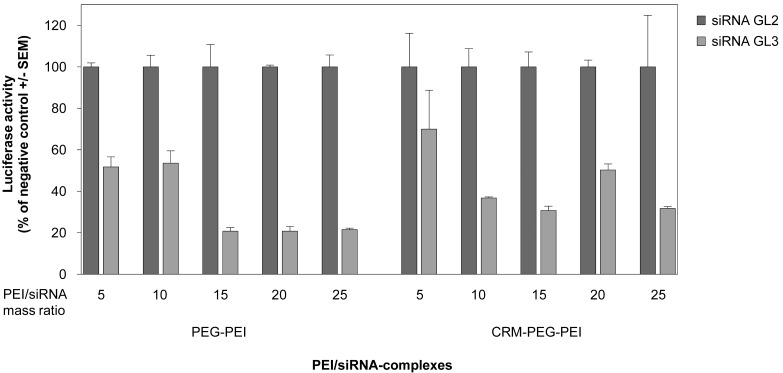
Determination of optimal (CRM197-)PEG-PEI/siRNA complex stoichiometries in an *in vitro* knockdown assay. The human glioblastoma cell line U87, transiently expressing luciferase, was transfected with PEG-PEI/siRNA complexes (left) or CRM197-PEG-PEI/siRNA complexes (right) at different (CRM197-)PEG-PEI/siRNA mass ratios, with complexes containing a non-specific siRNA (siRNA GL2; dark grey) or the luciferase-specific siRNA GL3 (light grey). Knockdown efficiencies after 48 h were determined by measuring luciferase activities in relative light units, and the respective negative controls are set to 100%.

When using cationic polymers, the application of optimal polymer/nucleic acid mixing ratios is critical for transfection efficiency. This optimal ratio can vary among different formulations as well as for different cell lines. Prior to *in vivo* experiments, we therefore investigated different mass ratios of the PEI-conjugates and siRNA as indicated in Figure 3 in order to determine the optimal ratio for siRNA-mediated gene knockdown in U87 cells. This was done by transient transfection of the glioblastoma cell line with a luciferase reporter gene prior to siRNA transfection after 24 h. For both PEI-conjugates a mass ratio of 15, equivalent to N/P ratio 115, resulted in best knockdown efficiencies. At this ratio, gene knockdown was 70–80% compared to the negative control (Figure 3). Higher mass ratios did not further improve the knockdown efficiency, and thus mass ratio 15 was used for the application of the complexes *in vivo*.

**Figure 4:**
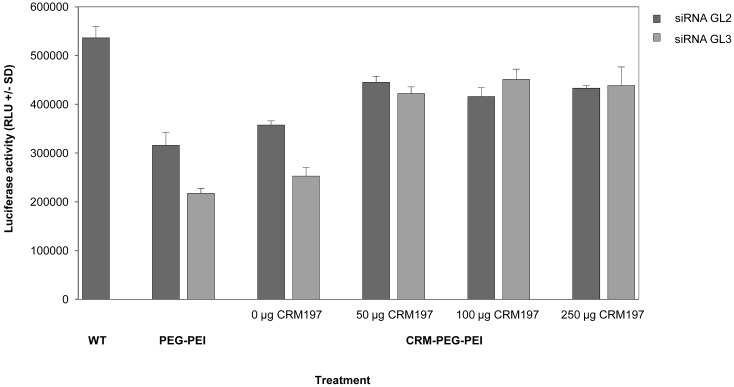
Competition of CRM197-mediated cellular uptake of CRM-PEG-PEI/siRNA complexes and siRNA-mediated knockdown in transiently luciferase expressing U87 cells. Comparison of luciferase activity in cells transfected with DNA (WT) and cells transfected with PEG-PEI/siRNA complexes or CRM197-PEG-PEI/siRNA complexes containing a non-specific siRNA (siRNA GL2; dark grey) or the luciferase-specific siRNA GL3 (light grey), respectively. CRM197-mediated uptake of CRM197-PEG-PEI/siRNA complexes was inhibited by addition of free CRM197 peptide 30 min prior to siRNA-targeting. Knockdown efficiencies after 48 h were determined by measuring luciferase activities in relative light units.

The CRM197-mediated specificity of the biological effects was determined in a competition experiment. To this end, various amounts of free CRM197 peptide were added to the cells 30 min prior to transfection. As shown in Figure 4, 50 μg free CRM197, as well as larger amounts, already completely abolished luciferase knockdown. In parallel, non-specific transfection effects were reduced upon CRM197 addition as well.

**Figure 5:**
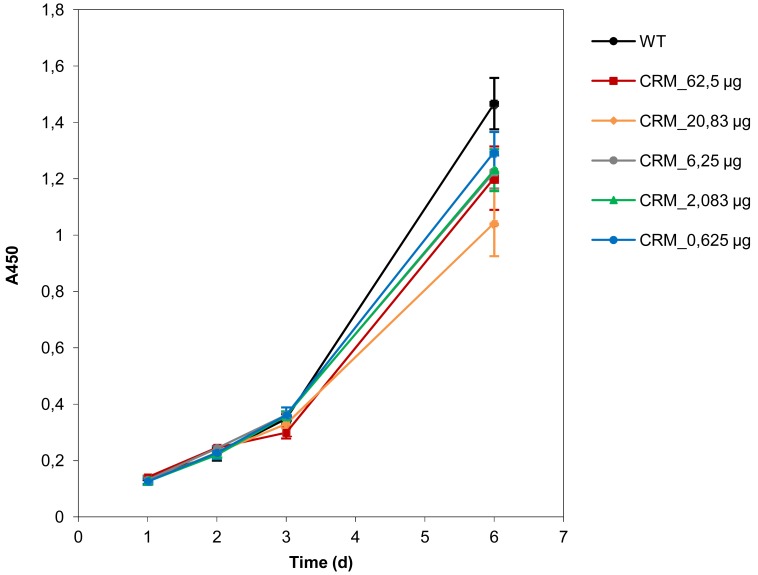
Proliferation of gliobastoma cells treated with CRM197 peptide. Cell proliferation of untreated U87 glioblastoma cells (WT; black line) compared to cells treated with different amounts of CRM197 (colored lines). The viability of cells was determined using the cell proliferation reagent WST-1 and the absorbance was measured at 450 nm.

Importantly, the determination of cell viabilities upon treatment with the targeting ligand CRM197 showed that the peptide alone did not mediate reduction of cell viability or proliferation, indicating that the gene knockdown is only due to PEI-mediated induction of RNA interference (Figure 5).

**Figure 6:**
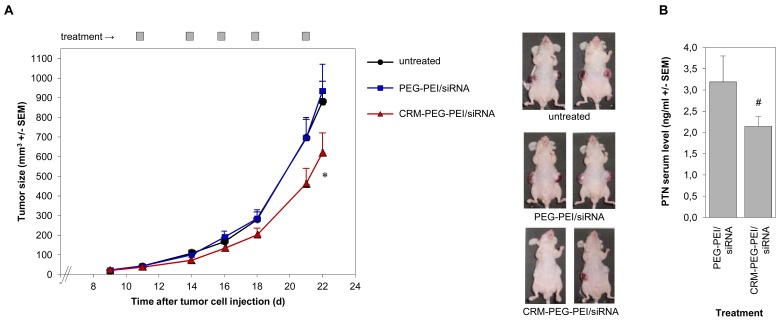
CRM197-PEG-PEI-mediated anti-tumor effects of CRM197-PEG-PEI/siRNA complexes targeting pleiotrophin (PTN). (**A**) After establishment of s.c. U87 glioblastoma xenografts, athymic nude mice were treated by intraperitoneal injection of PTN-specific CRM197-PEG-PEI/siRNA complexes (red triangles) or PEG-PEI/siRNA complexes (blue rectangles) at the time points indicated, or were left untreated (black circles). Left panel: quantitation of tumor sizes during the treatment (12 tumors per group; p < 0.02 between CRM-PEG-PEI/siRNA and PEG-PEI/siRNA or CRM-PEG-PEI/siRNA and untreated, respectively); right panel: pictures of representative mice from each group upon termination of the experiment. (**B**) PTN serum levels after termination of the experiment, as determined by ELISA (serum of six mice per group).

Finally, we investigated the PEI/siRNA-mediated knockdown of the tumor-relevant growth factor pleiotrophin (PTN) in an *in vivo* tumor model. After establishment of s.c. glioblastoma xenografts, we started the treatment of the mice by intraperitoneal injections of the polyplexes every 2–3 days. In addition to an untreated group, we directly compared mice treated with CRM197-PEG-PEI/siRNA complexes *vs.* PEG-PEI/siRNA complexes. Since the ligand CRM197 shows high affinity to its receptor, expressed by the tumor cells of the U87 xenografts, we expected preferred uptake of the CRM197-conjugated polyplexes as compared to PEG-PEI/siRNA complexes. In accordance with this assumption, the treatment with the targeted polyplexes resulted in reduced growth of the glioblastoma xenografts compared to untreated animals, while no antitumor effects were observed in the PEG-PEI/siRNA group [Figure 6(A)]. At the latest treatment time points, the tumor volumes of the CRM197-PEG-PEI treated mice were only about 60-70% of that of the control animals. This finding was supported by reduced PTN serum levels in the CRM197-PEG-PEI-treated mice as compared to the negative control, due to the reduced tumor mass and/or the PTN knockdown in the target cells [Figure 6(B)].

## 4. Discussion

Since the delivery of pharmaceutical substances to the target tissue is crucial for their activity, the development of drug targeting formulations is of high importance. Targeted delivery not only enhances the drug concentration at the site of action, but can also allow the administration of lower doses of the therapeutically active compound, thus reducing any unwanted side effects of the formulation. Drug targeting approaches are investigated for the delivery of substances to hardly accessible organ systems like e.g., the brain [31], or for the treatment of various diseases including tumors [32].

Gene therapy is a promising therapeutic approach, and the development and modification of various non-viral vectors is investigated to generate cell- or tissue-specific delivery systems. Modifications with ligands include peptides, sugars, antibodies and different small molecules. Here, we used the peptide CRM197 that was developed recently for the delivery across the blood-brain barrier [27] to generate a non-viral delivery system for tissue specific uptake of siRNA molecules. The conjugation of CRM197 to PEI F25-LMW was performed to achieve preferred uptake of the polyplexes by the cells of glioblastoma xenografts.

For efficient cellular uptake, non-modified polyplexes have to possess a net positive surface charge, but this positive charge can mediate non-specific uptake and is thought to be responsible for cytotoxic effects of the polymer [33-36]. The extent of the cytotoxicity highly depends on the structure and molecular weight of PEI. In general, high molecular weight branched PEI is more toxic than PEI with lower molecular weight and linear structure. In previous studies, we developed the low molecular weight branched PEI F25-LMW [11] and established it as a polymeric delivery system with high biologic activity and low cytotoxicity *in vitro* and *in vivo* [9,10,30,37].

For the non-toxic mutant of the diphtheria toxin, CRM197, a high biocompatibility was demonstrated [25], and the specific receptor-mediated uptake of a CRM197-conjugated protein by DTR has been successfully demonstrated in the brains of guinea pigs [26,38]. Although CRM197 possesses anti-tumor activity, which was shown to be due to the inhibition of the mitogenic activity of HB-EGF [39-42], our *in vitro* results suggest that this does not play a role at the CRM197 concentrations used here for the targeting of the polymer. Since the antitumor activity of CRM197 might be also immunological in nature [43], this will have to be addressed in subsequent studies by using complexes containing a negative control siRNA. The same is true for the effect of the PEG-PEI polymer, although in previous *in vivo* experiments we could demonstrate that the growth of tumors from mice treated with PEI/negative control siRNA complexes was identical to the growth of tumors from untreated animals [9,44,45]. The development of a cell-specific activity of nanoparticles relies on the one hand on the increase of their specificity by conjugation of target-specific ligands, and on the other hand on modifications that reduce unspecific interactions. From the literature and from our own experience, it is known that the reduction of unspecific interactions between the polyplexes and non-target tissues is necessary when working with PEI-based delivery systems [32,46]. Therefore, we performed PEGylation of PEI, which is an established modification, with the aim of influencing the chemical and pharmacokinetic properties of pharmaceutical substances [47]. In addition to the shielding of the positive surface charge, PEGylation reduces the formation of large aggregates of the polyplexes, which are not suitable for receptor-mediated endocytotic uptake [48,49]. In accordance with these considerations regarding the design of shielded nanoparticles for site selective drug targeting, we were able to demonstrate a reduction of the growth of s.c. tumor xenografts in the mice of the CRM-PEG-PEI/siRNA treatment group. Further optimization of the treatment regimens e.g., with regard to timing, amounts and mode of application are expected to lead to more profound antitumor effects.

## 5. Conclusions

In this work, the analysis of the PEI-conjugates revealed the biological activity of the targeted polyplexes. In accordance with previous studies, we could also show that the expression of the tumor-relevant growth factor PTN is rate-limiting for the growth of glioblastoma xenografts [20,21]. We establish the modification of PEI F25-LMW by CRM197-coupling as a novel non-viral delivery system suitable for the tissue-specific treatment of s.c. U87 glioblastoma xenografts. Upcoming studies will include the optimization of the treatment regimen, the administration of larger doses as well as the analysis of other modes of administration including i.v. injection. This will also include the exploration of the ligand-mediated trafficking of the nanoplexes across the blood brain barrier for the treatment of orthotopic glioblastomas.
